# Systematic identification of facility-based stillbirths and neonatal deaths through the piloted use of an adapted RAPID tool in Liberia and Nepal

**DOI:** 10.1371/journal.pone.0222583

**Published:** 2019-09-19

**Authors:** Blanche Greene-Cramer, Andrew T. Boyd, Steven Russell, Erin Hulland, Erin Tromble, Yulia Widiati, Sharad Sharma, Asha Pun, Denise Roth Allen, Emily Kainne Dokubo, Endang Handzel

**Affiliations:** 1 Emergency Response and Recovery Branch, Division of Global Health Protection, Centers for Disease Control and Prevention, Atlanta, Georgia, United States of America; 2 Health Section, UNICEF Liberia, Monrovia, Liberia; 3 Management Division, Department of Health Services, Kathmandu, Nepal; 4 Health Section, UNICEF Nepal, Kathmandu, Nepal; 5 Liberia Country Office, Division of Global Health Protection, Centers for Disease Control and Prevention, Monrovia, Liberia; Cincinnati Children’s Hospital Medical Center, UNITED STATES

## Abstract

Maternal, fetal, and neonatal health outcomes are interdependent. Designing public health strategies that link fetal and neonatal outcomes with maternal outcomes is necessary in order to successfully reduce perinatal and neonatal mortality, particularly in low- and middle- income countries. However, to date, there has been no standardized method for documenting, reporting, and reviewing facility-based stillbirths and neonatal deaths that links to maternal health outcomes would enable a more comprehensive understanding of the burden and determinants of poor fetal and neonatal outcomes. We developed and pilot-tested an adapted RAPID tool, Perinatal-Neonatal Rapid Ascertainment Process for Institutional Deaths (PN RAPID), to systematically identify and quantify facility-based stillbirths and neonatal deaths and link them to maternal health factors in two countries: Liberia and Nepal. This study found an absence of stillbirth timing documented in records, a high proportion of neonatal deaths occurring within the first 24 hours, and an absence of documentation of pregnancy-related and maternal factors that might be associated with fetal and neonatal outcomes. The use of an adapted RAPID methodology and tools was limited by these data gaps, highlighting the need for concurrent strengthening of death documentation through training and standardized record templates.

## Introduction

Each year 2.6 million children die within the first 28 days of life, known as the neonatal period; approximately 1 million die within the first day of birth and another 1 million die within the first week [1]. Neonatal deaths now account for nearly half of all deaths in children under five years, an increase from 40% in 1990, due to improvements in under-5 mortality while neonatal mortality remained stagnant [1]. An additional 2.6 million stillbirths (fetal death >28^th^ week of gestation) occur each year [2]. Mortality among babies in utero on or after 28 weeks (stillbirths) and in the first four weeks of life—a timeframe that includes both perinatal (≥28 weeks in utero to 7 days after birth [3]) and neonatal (≤28 days after birth)—results from a combination of medical causes, social factors, and system failures that vary by context [4,5]. Much of this mortality is attributable to the lack of quality maternal care before and during birth and neonatal care immediately after birth [1,6]. Improved care at birth has the potential to prevent almost half of all stillbirths in the intrapartum period as well as reducing preventable maternal and neonatal deaths [7]. Since maternal, fetal, and neonatal health outcomes are interdependent, it is crucial to develop public health solutions that better link fetal and neonatal health outcomes with maternal outcomes in order to reduce perinatal and neonatal mortality.

Complicating the lack of surveillance of stillbirths and neonatal deaths is the fact that many low- and middle-income countries struggling to reduce maternal and neonatal mortality rates lack systematic registration for pregnancies, births and deaths [8]. These countries also often do not have standardized data sources such as ward registers or logbooks with data on cause of death and potential aggravating factors for mothers and their babies. Additionally, there is a lack of consistent and comprehensive documenting, reporting, and reviewing of stillbirths and neonatal deaths that would allow for monitoring changes in burden [8]. Outcomes of mothers and their newborns also often go unrecorded, particularly when a woman goes through pregnancy and childbirth without any contact with a formal healthcare system [8]. Even when mothers seek and receive treatment, records are often incomplete and the information available about timing and cause of death and contributing or exacerbating factors is often insufficient to draw conclusions [2]. Fully capturing occurrences and assigning causes of stillbirths and neonatal deaths is not straightforward and requires use of multiple data sources, which can be challenging even if there is good documentation in facility records. All of these factors contribute to poor data quality, underreporting, and misclassification of stillbirths and neonatal deaths, suggesting that current global estimates for both stillbirth and neonatal death are likely underestimates [2].

Knowing the timing and causes of neonatal deaths in a country or geographic area is crucial to determining appropriate prevention strategies and interventions [2]. Similarly, differentiating stillbirths by timing facilitates tailored prevention and intervention resources. Antepartum stillbirths (fetal deaths occurring in the womb, before labor) often result from lack of antenatal care or inadequate management of maternal or pregnancy-related factors [5]. Knowledge of common maternal or pregnancy complications that precede stillbirths helps focus public health and clinical efforts to manage these complications. Intrapartum stillbirths (fetal deaths occurring during labor) may indicate complications during birth and a lack of access to or availability of quality emergency obstetric management and care [7].

An initial step in improving data on stillbirths and neonatal deaths is to utilize infrastructure for reporting pregnancy-related deaths, since early neonatal and stillbirth outcomes are linked to maternal outcome, and to focus on clinical facilities where there may be existing reporting protocols. In 2007, the University of Aberdeen’s Initiative for Maternal Mortality Programme Assessment (Immpact) developed a toolkit that provides a guide and tools for evaluating maternal mortality programs [9]. One of the tools, the Rapid Ascertainment Process for Institutional Deaths (RAPID), has been used in several settings to identify and collect unreported and misclassified pregnancy-related deaths from facility records [10,11]. In Haiti, RAPID resulted in the identification of 25% more pregnancy-related deaths than routine surveillance, with 23% previously misclassified as non-pregnancy-related [11]. RAPID differs from routine pregnancy-related death surveillance in that it requires review of all deaths of women of reproductive age rather than just those noted as pregnant. It also requires a review of registers from all wards where women of reproductive age may be admitted rather than just those in maternity wards, as well as death registers [11]. RAPID also categorizes pregnancy-related deaths by timing in relation to pregnancy: during pregnancy, during delivery, and within 42 days after termination of pregnancy. RAPID can also be used as part of a comprehensive surveillance evaluation to identify gaps in routine surveillance procedures and provide field training for surveillance staff on proper protocol for maternal mortality data collection. While the RAPID tool collects some information from facility registers related to neonatal outcomes, it is designed to focus on maternal outcomes. To date, no complementary tool has been developed to provide information about stillbirths and neonatal deaths that occur in facilities as well as integrates infant and maternal outcomes.

Ninety-nine percent of the burden of neonatal deaths occur in low and middle-income countries (LMICs), where pregnancy and childbirth can be life-threatening for mothers and their babies [1]. These dangers are compounded in fragile countries beset by humanitarian emergencies including Liberia and Nepal–the countries chosen for this study. Liberia has struggled with recurrent disease outbreaks following prolonged civil war, and the 2014–2015 Ebola outbreak overwhelmed already strained healthcare resources [12]. Estimates from 2016 showed that Liberia is once again struggling with high maternal mortality, (1072 deaths per 100,000 livebirths), neonatal mortality (26 deaths per 1000 births), and stillbirth rates (27 deaths per 1000 births) [13, 14]. Over the last several decades, Nepal has experienced cyclical natural disasters that prevent basic and healthcare infrastructure from fully rebuilding before subsequent disasters [15]. Washed out roads, destroyed bridges, and damaged facilities inhibit patients’ access to care as well as healthcare providers’ ability to administer timely and adequate care [15]. Nepal has had some success in reducing maternal and under-five mortality over the last few decades. However, as of 2016, maternal death rates, neonatal death rates, and stillbirth rates remained high (258 deaths per 100,000 live births, 21 deaths per 1000 live births, 18 deaths per 1000 births) [16, 17].

In 2017, the U.S. Centers for Disease Control and Prevention (CDC), the United Nation’s International Children’s Emergency Fund (UNICEF), and WHO developed a tool called Perinatal-Neonatal RAPID (PN RAPID), adapted from RAPID, to systematically identify and quantify facility-based stillbirths and neonatal deaths. The tool was also designed to provide a measure of standardization of surveillance of stillbirths and neonatal deaths and to more comprehensively link maternal outcome to fetal and neonatal outcomes. This study reports on the development and piloting of the PN RAPID tool to identify gaps in recording and reporting of stillbirths and neonatal deaths, particularly those related to timing and cause of death, and link to maternal outcomes in two countries: Liberia and Nepal.

## Methods

### Tool development

To develop complementary Perinatal-Neonatal tools to those used in RAPID for pregnancy-related deaths, we started by including basic information such as pregnancy outcome, gestational age at death for stillbirths or age in days for neonatal deaths, date of delivery, date of death, timing of death (antepartum, intrapartum, postpartum), fetal/neonatal complications, and suspected or final cause of death if death review or certificate was available. We also included demographics about the mother such as age, gravida and parity, maternal complications, and maternal outcome. In keeping with the RAPID methodology, we developed two excel forms to abstract these data–one for abstracting information from ward registers and the other for abstracting information from patient charts.

Through register review in Liberia, we identified key areas missing from most records and our abstraction forms that would be important for providing a comprehensive understanding of stillbirths and neonatal deaths. Therefore, before piloting in Nepal, we added fields for WHO evidence-based interventions newborns should receive in the first 24 hours of life [18], evidence or record of prenatal care visits, and information on pregnancy-related complications and maternal factors affecting poor fetal and neonatal outcomes, such as maternal HIV and syphilis testing and status. A list of variables abstracted in Liberia and Nepal are included in S1 Fig.

### Data abstraction

Over ten days in May 2017, four CDC and UNICEF personnel visited two urban referral hospitals (one general and one maternity) in one county in Liberia. Over ten days in August 2017, six CDC, UNICEF, and Nepal MoH personnel visited one urban maternity hospital and one general rural hospital in two districts in Nepal. All facilities visited were tertiary facilities.

Following a methodology adapted from maternal RAPID, the study team reviewed patient registers in each health facility for 2015 and 2016 for all wards where a neonate (age 0–28 days) might be admitted or a stillbirth might be documented. Wards included were maternity, obstetrics, delivery, emergency, surgery/operating, internal medicine, pediatrics, and neonatal intensive care unit (NICU). In Liberia, register data on stillbirths or neonatal deaths were captured using the PN RAPID register data capture form in Microsoft Excel (Microsoft Office 2013). In Nepal, register data were captured using the PN RAPID register data capture form in Open Data Kit (ODK) [19]. After identifying stillbirths or neonatal deaths through register review, abstractors then obtained and reviewed the corresponding medical records to confirm clinical history and gather additional data to determine timing (neonate vs stillbirth; antepartum vs intrapartum stillbirth, early vs late neonate) and cause of death. Maternal charts of mothers with stillbirths and early neonatal deaths found during register review were also reviewed. In facilities where a separate chart was maintained for deceased patients, staff reviewed charts of all death registers for the period of interest to identify any additional stillbirths or neonatal deaths that were not captured in the registers.

To determine a death as a stillbirth, we looked for documentation of gestational age, while for neonates we looked for documentation of age in days. If there was no documentation of gestational age or age since birth, the deaths were considered preliminarily unspecified. For each preliminarily unspecified death, the chart was reviewed for evidence of gestational age or age since birth. If there was no documentation of this information in the chart, the timing category of death (stillbirth or neonatal death) was finally listed as an unspecified stillbirth or unspecified neonatal death. Among all deaths categorized as neonatal deaths, those that occurred between days 8 and 28 were classified as late neonatal deaths while those that occurred between days 0 to 7 were classified as early neonatal deaths. Deaths that occurred on day 0 were closely examined during chart review for additional details such as the birth method, the mother’s health, etc. that might provide additional details to differentiate between intrapartum stillbirth or early neonatal death (live born but died shortly after birth). Among deaths classified as stillbirth, those listed as macerated stillbirth were classified as antepartum stillbirth, those listed as fresh stillbirth were classified as intrapartum stillbirth. If the suspected cause of death was documented in the register or chart, it was recorded as the suspected cause of death. If the suspected cause of death was not documented, physicians on the study team inferred the most likely suspected cause of death based on the signs, symptoms, complications, and clinical course described in the records. If there was no or limited information on suspected cause of death in the register or chart, the suspected cause of death was classified as unknown. Where a formal death review or death certificate was available as part of patient records, the final cause of death was recorded from these documents. The cause of death, whether suspected or final, was then categorized in accordance with WHO application of ICD-10 to deaths in the perninatal period (WHO ICD-PM) [20]. WHO’s ICD-PM is a standardized system used to classify stillbirths and neonatal deaths while linking them to contributing conditions in pregnant women.

### Data analysis

Data analysis was conducted using SAS version 9.4 (SAS Institute Inc., Cary, NC) and RStudio version 3.4.2. For both countries and across all examined facilities and both years, frequencies and percentages for the timing of neonatal deaths and stillbirths were determined based on indicators that were available in the registers. For Liberia, we compared the timing of death between the first day of life and days 2–7 to see if the proportion of categories of timing of deaths within the first week of life (the early neonatal period) differed significantly from what was expected based on UNICEF global data (50% for each the first day of life and days 2–7) [21]. While this comparison is not ideal because UNICEF data are community-based and ours is facility-based, it is the only one available and provides a standardized comparison. We also used Chi-square goodness-of-fit tests and Fisher’s exact tests to generate the frequencies of neonatal death timing categories to assess whether the distribution of timing of neonatal deaths differed significantly from what we would expect to find (38% in the first day of life, 38% between days 2–7, and 24% between day 8–28) [21]. These analyses were not possible in Nepal because of missing data.

To assess whether the top causes of death differed significantly between early neonatal age groups (≤ 1 day, days 2–7), Chi-square tests and Fisher’s exact tests were performed to compare proportions of causes of deaths. This analysis was only done in Liberia and not Nepal due to missing data.

Frequencies were run for location/ward of deaths, pregnancy-related complications and maternal factors related to poor fetal and neonatal outcomes (maternal HIV and syphilis testing and status) for Nepal as these questions were added during revisions to the tool after data collection in Liberia. For the same reason, frequencies of categories of pregnancy-related complications were also only calculated for Nepal.

### Ethical review

Because this study was an evaluation of public health surveillance program with de-identified data and no perceived ethical risk to patients, no informed consent was obtained. This evaluation received non-research determination from CDC’s Center for Global Health.

## Results

### Findings from the pilot test: Liberia

Pilot testing of the PN RAPID tools at two hospitals in Liberia identified 63 stillbirths and 263 neonatal deaths in facility registers during 2015–2016 (Table 1). Of the 63 stillbirths, six were classified as antepartum and six as intrapartum based on the information available in facility registers. For 51(81%) of the stillbirths, there was insufficient information in the registers to determine timing. Additionally, among stillbirths, there was no information available on cause of death or fetal complications in the registers to provide information on suspected cause of death.

**Table 1:** Stillbirths and neonatal deaths identified using PN RAPID in two facilities in Liberia.

	Observed n (%)	Expected n (%)
**Stillbirths (N = 63)**		
Antepartum death	6 (9.5)	31.5 (50)
Intrapartum death	6 (9.5)	31.5 (50)
Time of death unknown	51 (81.0)	-
**Neonatal deaths (N = 263)**		
≤ 1 day	133 (50.6)	100 (38)
2 to 7 days	79 (30.0)	100 (38)
8 to 28 days	26 (9.9)	63 (24)
Age at death unknown	25 (9.5)	-

Among the 263 neonatal deaths, 81% (212) were classified as early neonatal deaths (0–7 days) with 63% of all early neonatal deaths, and 51% of all neonatal deaths occurring within the first day of life and an additional 30% of all neonatal deaths occurring between 2–7 days. Just under ten percent of all neonatal deaths (n = 26) were classified as late neonatal deaths, occurring between 8–28 days. Register data was not sufficient to identify age at death for the remaining 25 neonatal deaths. Chi-square goodness-of-fit tests for only neonatal deaths with known time of death revealed that the timing of neonatal deaths in Liberia differed significantly from the proportions expected based on global data when categorized in two age groups (≤1 day vs. 2–7 days, p = 0.0002) and three age groups (≤1 day vs. 2–7 days vs. 8–28 days, p<0.0001), with higher than expected deaths within the first day of life.

Among the 212 identified early neonatal deaths, 193 were attributed to one of five causes: 86 deaths (40.6%) were from birth asphyxia, 41 (19.3%) due to disorders of newborn related to short gestation and low birth weight, 30 (14.2%) due to bacterial sepsis of newborn, 29 (13.6%) due to other conditions originating in perinatal period, and 7 (3.3%) due to respiratory condition of newborn, unspecified (Table 2). The remaining early neonatal deaths were distributed across 16 other ICD-10PM codes (S1 Table).

**Table 2:** Top five causes of neonatal death identified using PN RAPID in two facilities in Liberia.

	Age at death		
	≤ 1 day	2 to 7 days	Total
	n (%^†^)	n (%^†^)	n (%^†^)
1. Birth asphyxia	57 (42.9)	29 (36.7)	86 (40.6)
2. Disorders of newborn related to short gestation and low birth weight	29 (21.8)	12 (15.2)	41 (19.3)
3. Bacterial sepsis of newborn	16 (12.0)	14 (17.7)	30 (14.2)
4. Other conditions originating in the perinatal period	13 (9.8)	16 (20.3)	29 (13.7)
5. Respiratory condition of newborn, unspecified	6 (4.5)	1 (1.3)	7 (3.3)
Total	121 (91.0)	72 (91.1)	193 (91.0)*

^**†**^ Percentages out of total deaths, not just among the top 5 categories and thus do not sum to 100%. For neonatal deaths ≤1-day, other categories accounted for 9.0%; for neonatal deaths between 2–7 days, other categories accounted for 8.9%; overall other categories accounted for 9.0%.

*Fisher’s exact tests only among top 5 categories: p = 0.0901

### Findings from the pilot test: Nepal

Pilot testing of the tool at two hospitals in Nepal identified 377 stillbirths and 78 neonatal deaths in 2015–2016 from the registers (Table 3). Of the 377 stillbirths, 149 (39.5%) were classified as antepartum and 152 (40.3%) were classified as intrapartum. There was insufficient information in the registers to classify timing of death for the remaining 76 (20.2%) stillbirths. Similar to Liberia, there was a dearth of register data on cause of death or fetal complications to provide information on suspected cause of death among stillbirths. Among neonatal deaths, 46 (59.0%) were classified as early neonatal deaths (0–7 days), with 26 (33.3%) of all neonatal deaths with timing identified occurring within the first day of life and 20 (25.6%) of all neonatal deaths with timing identified occurring between 2–7 days. Only three (3.8% of all neonatal deaths) were classified as late neonatal deaths, occurring between 8–28 days. Information about infant age at death was missing for 29 (37.2%) neonatal deaths. Chi-square goodness-of-fit tests for only neonatal deaths with known time of death revealed that the timing of neonatal deaths differed significantly from the expected proportions when looking at three age groups: ≤1 day vs. 2–7 days vs. 8–28 days (p = 0.0039), with fewer than expected identified as occurring during the 8-28-day period and more than expected identified as occurring ≤1 day.

**Table 3:** Stillbirths and neonatal deaths identified using PN RAPID in two facilities in Nepal.

	Observed n (%)	Expected n (%)
**Stillbirths (N = 377)**		
Antepartum death	149 (39.5)	188.5 (50)
Intrapartum death	152 (40.3)	188.5 (50)
Time of death unknown	76 (20.2)	-
**Neonatal deaths (N = 78)**		
≤ 1 day	26 (33.3)	30 (38)
2 to 7 days	20 (25.6)	30 (38)
8 to 28 days	3 (3.8)	18 (24)
Age at death unknown	29 (37.2)	-

Among the 78 identified neonatal deaths, information on suspect or final cause of death was missing for 56 (71.8%) neonatal deaths (Table 4). For the 22 (28.2%) neonatal deaths where cause of death was available, the top five causes of neonatal death are reported in Table 4. The high numbers of deaths that lacked information on age at death or cause of death may have precluded both Chi-square goodness-of-fit and Fisher’s exact test from detecting differences in timing of neonatal deaths (early vs late neonatal deaths).

**Table 4:** Top five causes of neonatal death identified using PN RAPID in two facilities in Nepal.

Causes of neonatal deaths (N = 78)	n (%)
1. Disorders of newborn related to short gestation and low birth weight	7 (9.0)
2. Other infections specific to the perinatal period	5 (6.4)
3. Other problems with newborn	5 (6.4)
4. Other respiratory/cardiovascular disorders originating in the perinatal period	3 (3.8)
5. Other conditions originating in perinatal period	2 (2.6)
None recorded	56 (71.8)

Documentation of maternal STI testing and maternal complications was missing for a majority of deaths (Table 5). Of the 377 stillbirths, there was no information on HIV and syphilis testing status for 80% of deaths. More than 90% of stillbirths and neonatal deaths had no pregnancy-related complications recorded in the register or the data was missing in the abstraction forms. We found no information in registers of prenatal care documentation or of newborns regarding receipt of any of the WHO evidence-based interventions for the first 24 hours of life.

**Table 5:** Maternal STI testing status and maternal complications identified during register abstraction in two facilities in Nepal.

**Maternal STI testing status among stillbirths (N = 377)**	**n (%)**
Maternal HIV test status documented (N = 69)	
Test documented, negative test result	25 (36.2)
Not tested	18 (26.1)
Test documented, but no results provided	26 (37.7)
Maternal syphilis test status documented (N = 68)	
Test documented, negative test result	14 (20.3)
Not tested	25 (36.2)
Test documented, but no results provided	29 (42.0)
**Maternal complications among stillbirths and neonatal deaths (N = 455)**	**n (%)**
No complications recorded/missing	413 (89.8)
Obstetric complications	31 (6.7)
Hypertensive disorders	2 (0.4)
Abortive outcomes	2 (0.4)
Unanticipated complications	2 (0.4)

## Discussion

This is the first documented use of an adapted RAPID methodology and tools for surveillance of stillbirths and neonatal deaths in hospitals, and the first field implementation in fragile resource-limited settings. Full implementation of the adapted methodology and tools was limited by significant gaps in facility documentation for stillbirths and neonatal deaths.

Among all neonatal deaths reviewed in Liberia, a high proportion were early and occurred within one day of birth. This suggests preponderance of deaths are likely due to intrapartum complications and a lack of early care, particularly the WHO first 24-hour interventions which have been identified as being key evidence-based recommendations as guide health care professionals to reduce mortality during pregnancy, childbirth, postpartum, and newborn periods [18]. These results are in keeping with the finding that in Liberia where a high proportion of neonatal deaths within the first 24 hours identified birth asphyxia as the suspected cause of death [18]. Across the early neonatal period, the most common causes of death found in Liberia were birth asphyxia, complications of prematurity, and neonatal sepsis aligned with most common causes of early neonatal death around the world [8,18].

In Nepal, the proportion of neonatal deaths with missing data was much higher than in Liberia due to missing fields in registers as well as missing fields in the data abstraction forms. It was therefore difficult to draw conclusions about most common causes of neonatal death and if causes differed between early and late neonatal deaths, since there was missing documentation in almost three quarters of deaths.

Among stillbirths with timing documentation in Nepal, the same number were antepartum and intrapartum. A community-based study conducted in six representative districts in Nepal showed that almost three-quarters of stillbirths were intrapartum, which would indicate problems with care during birth [22]. The proportions found in these two tertiary facilities in Nepal through PN RAPID suggest a more even split, indicating gaps or inadequacies in antenatal care as well as problems with quality of care at birth, or that complications during birth could be managed more skillfully or with more resources. A comparison of proportions of timing of stillbirth was not possible in Liberia because of missing data in examined registers. However, in most LMICs two thirds of stillbirths are intrapartum (~60% in South Asia) [7]. It is possible the higher proportion of antepartum deaths found in this study were due to misclassification based on incomplete information.

In both countries, there was limited information on timing and cause of stillbirths, largely because these deaths are not routinely surveilled, and there is a lack of precise diagnostic information to aid in obtaining this information. Understanding timing of stillbirths could be improved with use of technology like ultrasound for prenatal visits and intrapartum fetal heart monitoring, or with low-technology modalities like fetal movement diaries, as well as through diagnosis and management of maternal pregnancy-related complications [23]. In cases of intrapartum stillbirth, improved emergency obstetric care may be beneficial [24]. Stillbirths need to be surveilled and reported in order to determine preventive measures.

In both Liberia and Nepal, the proportions of neonatal deaths across different categories was significantly different than the expected proportions based on UNICEF global data and we found higher proportions of death in the first day of life than expected. These findings may reflect poor documentation and reporting over time after a birth, or alternatively a potential bias of facility-based deaths being enriched for intrapartum deaths or neonatal deaths in the first day of life (high-risk births or delayed care seeking during delivery), since babies may be sent home after the first day and later die in the community. These findings reinforce the need to strengthen care immediately after birth using the WHO recommended interventions for the first 24 hours through improved training and supervision [18].

Using the revised tool in Nepal, the team examined registers recording stillbirths and neonatal deaths for maternal and pregnancy-related factors associated with stillbirths. The majority of entries listed no maternal complications, but it is not clear whether this was because there were no preceding complications present, if they were present but not diagnosed, or were diagnosed but not recorded in the medical record. Similarly, we found very limited documentation on HIV, syphilis and malaria testing, indicating a need for including testing and documentation in standardized protocols to improve diagnosis and treatment. There was no documentation in the registers of prenatal care visits in Nepal, information that should be routinely collected to allow for tracking of these quality of care indicators.

Maternal complications require diagnosis, which would be supported by routine prenatal care, yet we found no register entries with indicators for receipt of prenatal visits in Nepal. The importance of quality maternal and neonatal care cannot be overstated. Bhutta and colleagues estimated the high coverage (≥90%) of available quality interventions across four categories (integrated antenatal care, quality care at birth, essential newborn care, and care of small and ill newborn babies) could save 162,000 women, 816,000 stillbirths, 1.95 million neonates [25]. Incomplete documentation may be due to the absence of key information columns on standardized registers, insufficient staff to manage patient load and complete documentation, or lack of reporting to avoid blame.

The revised tool also allowed for examination of registers recording stillbirths and neonatal deaths for the presence or use of the WHO evidence-based interventions to be done in the first 24 hours of birth [18]. In the two hospitals visited, we found no systematic inclusion, or at least no documentation of inclusion in registers, of any of these interventions as part of routine post-birth care. The systematic documentation, surveillance, and quality review of the implementation of these interventions are paramount to improving neonatal outcomes [8,18,26], and including these interventions as indicators in registers may improve their documentation and perhaps their implementation. In both Liberia and Nepal, where high proportions of neonatal deaths are early, assuring evidence-based interventions are performed may have a significant impact on neonatal mortality in these countries.

One of the benefits of RAPID is that it facilitates the identification of deaths in all wards of the facility, rather than only in those where routine reporting is done. In Liberia, many stillbirths and neonatal deaths were not found in ward registers, but in death registers and admission registers. Similarly, in Nepal, a number of stillbirths and neonatal deaths were found outside of wards where one might expect such deaths to be recorded, including in the operating theater, as well as in the general admissions and discharge register. While stillbirths and neonatal deaths require reporting in Liberia and Nepal and protocols for reporting and review have been developed, implementation has been incomplete and inconsistent. Because stillbirths are distinct from neonatal deaths, it is important that surveillance be conducted in all wards of the hospital and using all registers in which deaths are recorded, and that definitions of stillbirths as well as late neonatal deaths (i.e., neonatal death is up to 28 days, even if the baby had been discharged home) be standardized and disseminated.

## Limitations

There are several limitations to this study. First, due to time constraints and challenges locating patient charts, the team was unable to abstract additional stillbirth and neonatal death data or any maternal data from patient medical records in Liberia and Nepal. The effect of this was two-fold. Had we been able to complete the review of any additional data provided in medical records that was not available in the registers we might have been able to reduce the number of unspecified deaths and present a more representative estimate of stillbirths and neonatal facility deaths by timing. Secondly, there may have been deaths that were not documented in the registers that we only would have found in the medical records, so without the complete review our results may be underestimating the true burden.

Second, the numbers and causes of death for stillbirths and neonatal deaths identified through the use of PN RAPID in Liberia and Nepal only reflect those found in registers. We did not include the stillbirths and neonatal deaths collected from patient charts because we only had access to a small portion due to challenges with location of charts and limited time. The charts we had access to were not systematically selected and could not be assumed to be representative. Inability to verify timing of deaths may have resulted in intrauterine deaths that occurred before 28 weeks that were classified as stillbirths, intrapartum stillbirths that were classified as early neonatal deaths, and neonatal deaths of unknown age that could not be categorized because the information was missing. Similarly, we occasionally found live births that had gestational ages listed even as late as 53 weeks, and some stillbirths with variable gestational ages. Without chart reviews to verify these findings, birth classification and timing were based on the most comprehensive information available from the registers.

There were some challenges to using the tool successfully in both Liberia and Nepal. Using available data sources in visited hospitals, it was often difficult to differentiate between stillbirths and neonatal deaths, in part because of overlap in accepted definitions of these three categories and inconsistent recording conventions. Among stillbirths, there was the additional challenge of trying to determine whether the medical register documentation of antepartum versus intrapartum was accurate and whether using “macerated” and “fresh” respectively as proxies for these two categories is valid, given the dearth of information on timing captured in facility registers. This may have contributed to misclassification between these categories. Going forward, more precisely differentiating these deaths by timing is important, because it allows for identification of most common causes of death for each period to enable better planning for prevention and response, both for staff training and in facility protocols and infrastructure.

Lack of medical record review also precluded abstracting information on maternal and pregnancy-related factors that may have influenced fetal and neonatal outcomes, as well as information on use of the WHO first 24-hour interventions. This information is not included in indicators in standardized registers in either country, so absence from the registers may mean that while it was not recorded in the register, it may have been recorded elsewhere. The information sought is important for routine surveillance of antenatal, birth, and postpartum care for both the mother and baby, and thus should be included in registers to improve surveillance as well as act as a “checklist” for implementation of evidence-based interventions to improve neonatal outcomes.

Additionally, reviewed facilities in Liberia and Nepal were tertiary care facilities able to provide comprehensive emergency obstetric care and may have a different distribution of cause of death or mortality rate than other facilities in these countries. Furthermore, these facilities were all selected purposively and thus the findings from these facilities cannot be generalized to populations seeking care as a whole in Liberia and Nepal.

## Conclusions

Findings from the pilot test of PN RAPID demonstrated that the tools hold promise for use in low-resource settings and could potentially be used in conjunction with RAPID to identify both stillbirths and neonatal deaths in facilities. In addition to helping establish protocols for identifying and reviewing stillbirths and neonatal deaths, PN RAPID pilot testing showed the need for enhanced pregnancy registration and facility-based pregnancy documentation in patient records to facilitate identification, timing, and causes of neonatal deaths and stillbirths. It showed the need to include critical predictors of fetal and neonatal outcomes in registers to facilitate their inclusion in routine surveillance. Pilot testing also highlighted the need to strengthen death registration and death review to provide more complete information about causes of stillbirths and neonatal deaths as well as major contributing factors. Pilot testing also highlighted the need to improve documenting maternal health status and maternal complications in stillbirth and neonatal records in order to link maternal with fetal and neonatal outcomes. Our finding that most neonatal deaths occurred in the early neonatal period, highlights the importance incorporating surveillance indicators to capture implementation of WHO’s first 24-hour interventions as a way to support the implementation and evaluate the impact of these interventions on neonatal outcomes in facilities.

The purpose of RAPID is not to identify final cause of death; the final determination of cause of death has to be done external to the RAPID process. However, suspected cause of death can be inferred using the patient charts. In Liberia, patient charts were not easily accessed, and thus complications listed in registers were used as a proxy for cause of death. Documentation of relevant complications and other distinguishing factors is needed in both the registers and charts for situations when registers cannot easily be linked to charts. Similarly, although most neonatal mortality information can be garnered from maternal charts, challenges with linkage were common and thus identifying key maternal variables to include in the neonatal charts is of great importance. While we still encourage stronger programming for the immediate neonatal period due to an unequal burden in deaths seen during the first day of life, we must equally focus on improving data collection and documentation to improve evidence.

In the future in Liberia and Nepal, PN RAPID could be used periodically, for example every 6 to 12 months, as a standard monitoring tool for routine facility-based surveillance since it emphasizes review of other ward registers and charts of deaths among neonates and stillbirths providing a more comprehensive capture of these deaths in facilities. Recommended frequency of use depends on factors such as existing quality of data, availability of staff, number of facilities/regions included, and other potentially competing priorities. Ongoing use of PN RAPID could help monitor improvements in reporting as well as provide additional details of location and timing of facility-based stillbirths and neonatal deaths in fragile and low-resource settings. Repeated use would help sensitize healthcare providers and public health staff to a key set of indicators for stillbirths and neonatal deaths but would also require commitment to systematically include these indicators in registers and charts as well as developing and maintaining a system for organizing and storing patient charts. While standardized registers and charts were said to have been developed, these were not present in the field and were missing some of the key pieces of information needed to link maternal with fetal and neonatal outcomes. While human resource intensive, improving routine data quality and introducing periodic monitoring through PN RAPID would be mutually reinforcing and provide high-quality data to help improve the management of pregnant women, childbirth, and neonatal care.

The PN RAPID tool helps to illustrate the link between maternal factors and fetal and neonatal outcomes, which supports the need for better harmonization and integration between maternal and perinatal health, both in terms of surveillance and intervention. PN RAPID is a first step in capturing stillbirths and neonatal deaths. It could then be used in conjunction with other methods, such as formal neonatal death review, as is being rolled out in Liberia and Nepal, as well as community-based quantitative and qualitative evaluations such as verbal autopsy, by health practitioners and administrators to more comprehensively and accurately measure, understand and address stillbirths and neonatal mortality in fragile countries.

## Supporting information

S1 Fig List of variables extracted using PN RAPID in Liberia and Nepal. (DOCX)

S1 Table Causes of neonatal death in two facilities in Liberia by age at death. (DOCX)
