# Supplementary material for: Systematic identification of facility-based stillbirths and neonatal deaths through the piloted use of an adapted RAPID tool in Liberia and Nepal
Source: PLoS One. 2019 Sep 19;14(9):e0222583. doi: 10.1371/journal.pone.0222583 (PMC6752757; doi:10.1371/journal.pone.0222583)
Supplement: S1 Fig — (DOCX) [file pone.0222583.s001.docx]

**S1 Figure: List of variables extracted using PN RAPID in Liberia and Nepal**

| **Variables** | **Liberia** | **Nepal** |
| --- | --- | --- |
| Facility ID | x | x |
| ID number | x | x |
| Maternal age | x | x |
| Delivery Date | x | x |
| Gestational age at birth | x | x |
| Maternal complications (antepartum, intrapartum) | x | x |
| Type of delivery (SVD, AVD, CS) | x | x |
| Type of birth | x | x |
| Date of death | x | x |
| Birthweight (kg) | x | x |
| APGAR score | x | x |
| Maternal outcome (living, transferred, dead) | x | x |
| Neonatal complications (Y/N) | x | x |
| Perinatal/Neonatal outcome (died antepartum, died intrapartum, died postpartum) | x | x |
| Observations | x | x |
| Date of last admission | x | x |
| Chief complaint | x | x |
| Clinical diagnosis (at admission; final) | x | x |
| Neonatal complications recorded (described) | x | x |
| Cause of death from death certificate (if available) | x | x |
| Evidence of status of death (describe) | x | x |
| Sources of information used to classify death (in-patient case notes, neonate register, death register, operating theater register, out-patient records, death certificate, other (specify) | x | x |
| Procedures performed (late cord clamping, skin to skin contact within 1 hour, initiation of breastfeeding, Vitamin K prophylaxis) |  | x |
| Evidence of prenatal care visits (Y/N) |  | x |
| Pregnancy-related complications (describe) |  | x |
| Maternal HIV testing and status |  | x |
| Maternal syphilis testing and status |  | x |
| Maternal complications (describe) |  | x |
