# Supplementary material for: Systematic identification of facility-based stillbirths and neonatal deaths through the piloted use of an adapted RAPID tool in Liberia and Nepal
Source: PLoS One. 2019 Sep 19;14(9):e0222583. doi: 10.1371/journal.pone.0222583 (PMC6752757; doi:10.1371/journal.pone.0222583)
Supplement: S1 Table — (DOCX) [file pone.0222583.s002.docx]

**S1 Table: Causes of neonatal death in two facilities in Liberia by age at death**

| **Cause of death among livebirth neonates based on**  **ICD-PM coding** | **Age at Death (days)** | | | | |
| --- | --- | --- | --- | --- | --- |
|  | **0 – 1** | **2 – 7** | **8 – 28** | **Unknown** | **Total** |
|  | **n (%)** | **n (%)** | **n (%)** | **n (%)** | **n (%)** |
| 1. Birth asphyxia | 57 (42.9) | 29 (36.7) | 0 (0.0) | 7 (28.0) | 93 (35.4) |
| 2. Disorders of newborn related to short gestation and LBW | 29 (21.8) | 12 (15.2) | 2 (7.7) | 6 (24.0) | 49 (18.6) |
| 3. Bacterial sepsis of newborn | 16 (12.0) | 14 (17.7) | 11 (42.3) | 5 (20.0) | 46 (17.5) |
| 4. Other conditions originating in perinatal period/unknown | 13 (9.8) | 16 (20.3) | 6 (23.1) | 1 (4.0) | 36 (13.7) |
| 5. Respiratory condition of newborn, unspecified | 6 (4.5) | 1 (1.3) | 0 (0.0) | 3 (12.0) | 10 (3.8) |
| 6. Hydrocephalus, unspecified | 2 (1.5) | 0 (0.0) | 0 (0.0) | 0 (0.0) | 2 (0.8) |
| 7. Respiratory distress of newborn | 2 (1.5) | 0 (0.0) | 0 (0.0) | 0 (0.0) | 2 (0.8) |
| 8. Neonatal aspiration | 2 (1.5) | 1 (1.3) | 0 (0.0) | 1 (4.0) | 4 (1.5) |
| 9. Cardiovascular disorders originating in perinatal period | 2 (1.5) | 0 (0.0) | 0 (0.0) | 0 (0.0) | 2 (0.8) |
| 10. Tetanus neonatorum | 1 (0.8) | 1 (1.3) | 2 (7.7) | 0 (0.0) | 4 (1.5) |
| 11. Neonatal hypoglycemia | 1 (0.8) | 2 (2.5) | 1 (3.8) | 0 (0.0) | 4 (1.5) |
| 12. Other specified congenital infectious and parasitic diseases | 1 (0.8) | 1 (1.3) | 0 (0.0) | 0 (0.0) | 2 (0.8) |
| 13. Disorders of newborn related to slow fetal growth and fetal malnutrition | 0 (0.0) | 0 (0.0) | 1 (3.8) | 1 (4.0) | 2 (0.8) |
| 14. Birth injury to skeleton | 1 (0.8) | 0 (0.0) | 0 (0.0) | 0 (0.0) | 1 (0.4) |
| 15. Diabetes mellitus neonatal | 0 (0.0) | 1 (1.3) | 0 (0.0) | 0 (0.0) | 1 (0.4) |
| 16. Other perinatal digestive system disorders | 0 (0.0) | 1 (1.3) | 0 (0.0) | 0 (0.0) | 1 (0.4) |
| 17. Other perinatal hematological disorders | 0 (0.0) | 0 (0.0) | 1 (3.8) | 0 (0.0) | 1 (0.4) |
| 18. Congenital anemia | 0 (0.0) | 0 (0.0) | 1 (3.8) | 0 (0.0) | 1 (0.4) |
| 19. Disorders of newborn related to long gestation | 0 (0.0) | 0 (0.0) | 1 (3.8) | 0 (0.0) | 1 (0.4) |
| 20. Newborn affected by complications of placenta, cord and membranes | 0 (0.0) | 0 (0.0) | 0 (0.0) | 1 (4.0) | 1 (0.4) |
| **Total** | 133 (50.6) | 79 (30.0) | 26 (9.9) | 25 (9.5) | 263 |
